# Stress, Social Support, and Resilience in Younger Rural Women: A Structural Equation Model

**DOI:** 10.3390/healthcare9070812

**Published:** 2021-06-28

**Authors:** Laurie S. Abbott, Lucinda J. Graven, Glenna Schluck, Krystal J. Williams

**Affiliations:** 1 College of Nursing, Florida State University, Tallahassee, FL 32306, USA; lgraven@fsu.edu (L.J.G.); glenna.schluck@fsu.edu (G.S.); 2 College of Pharmacy and Pharmaceutical Sciences, Florida Agricultural & Mechanical University, Tallahassee, FL 32307, USA; krystaljwilliams@hotmail.com

**Keywords:** cardiovascular disease, resilience, rural, social support, stress

## Abstract

Cardiovascular disease is a global public health problem and leading cause of death. Stress is a modifiable cardiovascular disease risk factor. The objectives of this study were to examine whether stress was a predictor of resilience among rural younger women and to explore whether social support mediated the relationship between acute stress and resilience and between chronic stress and resilience. The study had a cross-sectional, descriptive design. A total of 354 women were randomly recruited in the rural, southeastern United States. Survey instruments were used to collect data about acute stress, chronic stress, social support, and resilience. A structural equation model was fit to test whether social support mediated the relationship between perceived stress and resilience and between chronic stress and resilience. Chronic stress predicted family and belongingness support and all the resilience subscales: adaptability, emotion regulation, optimism, self-efficacy, and social support. Acute stress predicted the self-efficacy subscale of resilience. Family support partially mediated the relationship between chronic stress and self-efficacy. Belongingness support partially mediated the relationships between chronic stress and the social support subscale of resilience.

## 1. Introduction

Cardiovascular disease is a global public health problem and leading cause of death throughout the world [1,2,3]. The major risk factors for cardiovascular disease are smoking, high plasma cholesterol levels, physical inactivity, obesity, and diabetes [2]. However, stress is an established and underrecognized risk factor of cardiovascular disease [4,5,6]. Perceived stress and having lower socioeconomic status activate the amygdalar region of the brain and triggers increased bone marrow activity, arterial inflammation, and future cardiovascular events [6,7]. Furthermore, people experiencing acute stress and those with chronic stress are more likely engage in health behaviors that increase their overall cardiovascular disease risk, including smoking, physical inactivity, and excessive alcohol intake [8]. Persistent, or chronic, stress increases the prevalence of coronary artery plaque as well as metabolic syndrome, which is a significant predictor and modifiable risk factor of coronary heart disease, stroke, diabetes, and CVD mortality [9,10,11,12].

The prevalence of these chronic conditions contributes to worse cardiovascular disease outcomes among both men and women with lower socioeconomic status living in remote and underserved areas of the southern United States [13]. In fact, the age-adjusted mortality rates reported for cardiovascular disease are higher among people living in rural areas compared with large metropolitan areas due to the economic slowdown, limited access to health care, and higher rates of determinants that increase overall cardiovascular disease risk [14]. Furthermore, rural, southern populations have disproportionately higher cardiovascular disease risk, morbidity, and mortality [15,16,17,18,19]. Rural, southern residents are also less likely to be physically active and more likely to be diagnosed with and managing co-morbidities of cardiovascular disease, including diabetes, hypertension, and obesity [16,17].

For women, cardiovascular disease is often under-diagnosed and undertreated, even though it has been recognized as a leading cause of death for women worldwide [20,21]. As the global population increases and ages, women will comprise the majority of people older than 65 years of age by 2030, and the associated increase in prevalence of cardiovascular disease for women after menopause will likely exceed the prevalence for men [22,23]. In addition to the other factors, such as diet, physical activity, and weight, stress is also a modifiable contributory CVD risk factor in women of all ages [24,25]. Perceived acute stress is described as the degree that a person appraises as being stressful and involves feelings of unpredictability and lack of control [26]. Women who have high stress levels are more likely to be younger, divorced or separated, and overweight or obese, and they are more likely to self-identify as being African American, smoke, have hypertension, and have worse overall cardiovascular health compared with those with lower stress levels [8,13,27,28].

Reactions to stressful stimuli can differ. For example, in response to stress, some people develop posttraumatic stress disorder and severe depression while others show resilience to stress by displaying no significant psychological signs [29]. Serving as a stress buffer and protective factor, resilience is defined as the successful adaptation to conditions of stress, trauma, and adversity that facilitates avoidance of mental disorders such as depression and anxiety [30,31]. Protective factors associated with resilience contain internal aspects such as attitudes and beliefs as well as external facets such as individual perceptions of having social support [32]. Relevant abilities associated with resilience in individuals include adapting to changing conditions and new circumstances, maintaining control over emotional reactions that affect decision-making, having an optimistic outlook on the future, believing that difficult situations can be handled in a manner that facilitates success, and being able to form supportive relationships with others [32]. Psychological resilience determines cardiometabolic health through direct linkages with stress, cortisol levels, and the severity of metabolic syndrome [33]. The higher the level of resilience, the inversely lower the levels of stress, cortisol, and metabolic syndrome severity [33,34]. These linkages between high resilience and better cardiometabolic health may be independent of a stress-buffering effect [33].

The relationship between stress and resilience is complex. However, social support can strengthen individual resilience, which can buffer the effects of stress, especially for women [35]. Theoretical conceptions of social support are described as being socially connected with others such as family and friends, having significant social relationships, and perceiving a sense of belongingness within a group [36]. Social support may facilitate resilience in response to stress through brain pathways such as the hypothalamic-pituitary-adrenocortical (HPA) system [37] and can positively influence an individual’s response to stress and reduce disease risk [31]. For example, support from significant others, family members, and social groups is related to lower rates of CVD-related conditions, including diabetes, hypertension, and stroke [36]. Social support can also facilitate improvements in other CVD risk factors, such as increased physical activity levels [38].

This study was guided by concepts from the Social Networks and Social Support model and theories of Resilience. The Social Networks and Social Support model posits that social support from being in reciprocal relationships with others has a positive impact on physical and emotional health by meeting basic human needs for companionship and belongingness [39]. The framework explains that as people experience acute stress, having social support from family and belongingness in social groups increases the ability to cope, which buffers the effects of stress and reduces health consequences and the development of chronic stress. Theoretical constructs of resilience include the phenomena of persisting, adapting, coping, and exhibiting positive outcomes despite serious threats by responding to adversity without disruptions in personal functioning [40,41]. Resilience involves protective factors that cultivate healthy personality traits and promote positive health outcomes [42]. Guided by these theoretical principles, the purposes of this study were twofold. First, we sought to examine whether stress was a predictor of resilience among younger women living in the rural, southeastern United States and, second, to explore whether social support from family and friends, family support and belongingness support, mediated the relationship between acute stress and resilience and between chronic stress and resilience.

## 2. Materials and Methods

This cross-sectional, descriptive study explored stress, social support, and resilience factors among a representative random sample of women living in a rural region of the southeastern United States. The inclusion criteria required that participants self-identified as being female, were between 18 and 44 years of age, inclusive, were not currently pregnant, and were residents of a rural region in the southeastern United States. The sample size (*n* = 354) was estimated by entering the population of women, aged 18–44 (*n* = 4540), residing in the targeted area [43], a 95% confidence level, and a confidence interval of 5 into a sample size calculator [44]. This study received approval from an institutional review board at the Florida State University and a state-level review board. Participants were randomly recruited from public areas, such as the rural county public libraries, neighborhoods, and parking lots. After being screened for inclusion criteria, those eligible and willing to participate received an informed consent form that was discussed and signed prior to data collection using self-reported survey instruments. The study participants received a $15 gift card to a nearby superstore after completing the surveys. The study was conducted from July 2019 until May 2020.

### 2.1. Measures

A sociodemographic survey was administered to collect information about participant socio-demographic characteristics, location of residence, financial insecurity, such as difficulty paying for food and housing, and the presence of minor-aged children living in the home. Data were collected from all study participants during one data collection point. For this study, acute stress was measured using the “Perceived Stress Scale” [45]. The instrument is a 10-item, 5-point Likert scale that had excellent internal consistency (α = 0.84–0.86). The measure includes questions regarding feeling and thoughts experienced during the last month about being upset, stressed, and angry. The response options range from “Never” (0) to “Very Often (4), and higher scores are associated with higher levels of acute stress.

The “Chronic Stress Index” is 30-item Likert scale-type tool that measures chronic stress and has excellent internal consistency associated with the categorical subscales: neighborhood physical (α = 0.69) and social environment (α = 0.77), safety (α = 0.84), everyday unfair treatment (α = 0.77), financial vulnerability, and numerical count of major life events [46]. The answer options range from “Disagree” (1) to “Agree” (5) for social and physical environmental issues and “Never” (1) to “Always” (5) for safety and unfair treatment items. The answer options were used in response to statements about environmental factors, such as the presence of pollution, loud noises, and theft, as well as personal perceptions of safety and treatment by others. The financial vulnerability questions asked how long the current standard of living could be maintained following loss of income with answer options ranging from “More than a Year” (1) to “Less than One Month” (5). There were eight items in the Major Life Events subscale that had “Yes” or “No’ responses to serious life events such as serious illness or injury, loss of loved one, or divorce from a spouse or partner within the last year. For each of the subscales, higher scores indicate higher levels of chronic stress.

Social support was measured using the “Family and Belongingness Social Support Measure” [36]. The instrument has subscales for family belongingness and social support. The survey items for the family belongingness subscale included talking and socializing daily with family, and the social support subscale measured the available support from friends, neighbors, and participation in organized group activities. The 6-item, 4-point Likert scale has excellent internal consistency for the family belongingness subscale (α = 0.76) and acceptable internal consistency for the social support subscale (α = 0.70). The instrument includes ratings ranging from “Never” (1) to “Always” (4) with higher total scores associated with higher levels of social support.

The “Five-by-Five Resilience Scale” is a 25-item, 5-point Likert scale instrument that had excellent overall internal consistency (α = 0.93) as well as within each of the five subscales, which were Adaptability (α = 0.86), Emotion Regulation (α = 0.90), Optimism (α = 0.92), Self-efficacy (α = 0.85), and Social Support (α = 0.88) [32]. The items consist of statements about adapting to new situations, keeping emotions under control, feeling comfortable around people, etc. The response choices range from “Very Inaccurate” (1) to “Very Accurate” (5). The higher the score, the higher the level of resilience.

### 2.2. Analytic Strategy

**Figure 1:**
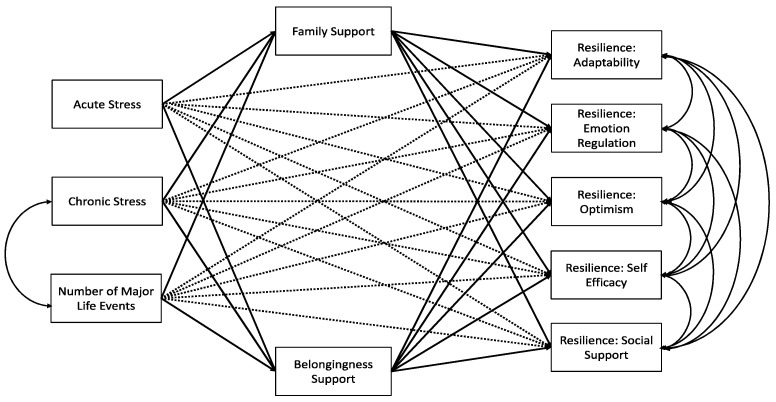
Original path analysis model examining family support and belongingness support as mediators of the relationship between chronic stress and resilience and between acute stress and resilience.

A structural equation model was fit to test whether family and belongingness support mediated the relationship between perceived stress and resilience and between chronic stress and resilience. A structural equation modeling framework was chosen because it simultaneously estimates all model coefficients and is considered an effective tool for examining mediation [47]. Acute stress is calculated as the sum of all items on the scale. Two subscales for the family and belongingness social support measure were included in the model as mediator variables: one each for family support and belongingness support. Family support and belongingness support were calculated as the mean of the three items in each subscale. The composite score was calculated as the mean of the six items [36]. Five subscales of resilience were included in the model as outcomes. The five subscales of resilience were each calculated as the mean of the five items in each subscale [32]. Chronic stress is composed of five subscales and the number of major life events experienced in the past 12 months. Each subscale is calculated as the average of the items in the subscale and major life events in the past 12 months is a count of the number of major life events. We calculated a composite score for chronic stress by calculating z-scores for each subscale and averaging the z-scores [46]. The chronic stress composite score and the number of major life events experienced in the past 12 months were included in the model. Additionally, the covariance between the chronic stress composite score and the number of major life events in the past 12 months was included as these are calculated from the same scale. The fitted model is depicted in Figure 1. Dashed lines in the figure will have insignificant coefficients if family support and belongingness support completely mediate the relationships depicted. If these paths have significant coefficients in addition to significant coefficients through the mediating variable, then the variable is a partial mediator.

Coefficients were estimated using maximum likelihood estimation with standard errors robust to non-normality. The comparative fit index (CFI) [48] and standardized root mean square residual (SRMR) [49] are reported as model fit indices. CFI values above 0.90 and SRMS values below 0.05 indicate adequate model fit [49,50]. Coefficients are reported for all paths in tabular format along with standard errors and associated *p*-values. Direct and indirect effects are also reported for completeness of the reader’s ability to inspect the effect size estimates for each relationship. Covariances are not reported but were modeled in the structural equation model mediation model. There were no missing data points in the variables of interest for this study.

## 3. Results

The participants (*n* = 354) who completed the study were primarily single (*n* = 239, 67.5%), Black (*n* = 289, 81.6%), and not Hispanic (*n* = 343, 96.9%). Their ages ranged from 18 to 44 with an average age of 32.7 years (SD = 7.9). Approximately half (*n* = 169, 47.7%) had a high school diploma or GED and 28.8% (*n* = 102) had gone to college for some number of years. Participants had between 0 and 8 children, with a median of 1 child (M = 1.5, SD = 1.5). More than two-thirds (*n* = 244, 68.9%) had children younger than 18 years old living in the home. Almost three-quarters (*n* = 256, 72.3%) of participants reported financial insecurity, such as having difficulty paying for food and housing with just their income and no other assistance. Participant demographics are displayed in Table 1.

Participants reported fewer than two major life events in the past 12 months on average (M = 1.8, SD = 1.8), above average family support (M = 3.5, SD = 0.7), average belongingness support (M = 2.7, SD = 0.7), and above average resilience (Adaptability: M = 3.6, SD = 0.7; Emotional Regulation: M = 3.0, SD = 0.7; Optimism: M = 3.7, SD = 0.9; Self-efficacy: M = 3.7, SD = 0.8; Social Support: M = 3.8, SD = 0.8). Additionally, participants reported low to moderate acute stress (M = 19.19, SD = 6.12) and average chronic stress (M = 0, SD = 0.68). They reported average chronic stress about neighborhood physical environment (M = 2.3, SD = 0.80) and safety (M = 2.34, SD = 1.34), below average chronic stress about neighborhood social environment (M = 2.0, SD = 1.18) and events of unfair treatment (M = 1.98, SD = 0.93), and above average chronic stress about financial vulnerability (M = 3.39, SD = 1.57).

**Figure 2:**
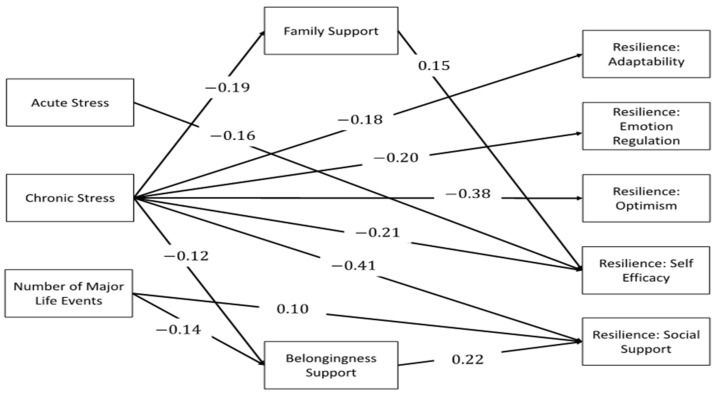
Path analysis model examining family support and belongingness support as mediators of the relationship between chronic stress and resilience and between acute stress and resilience. Correlations and paths with insignificant *p*-values are not depicted.

The fitted model adequately fit the data (CFI = 0.916, SRMS = 0.041). Due to the complexity of the model, the full fitted model is depicted in Figure 1, and a figure with only the statistically significant paths is shown in Figure 2.

All model coefficients, their standard errors, and associated *p*-values are reported in Table 2. Table 3 contains the estimates for the direct and indirect effects for the relationships in the model. All reported model estimates are standardized estimates. Chronic stress was a significant predictor of family support (b = −0.19, *p* = 0.001), belongingness support (b = −0.12, *p* = 0.035), and all resilience subscales (Adaptability: b = −0.18, *p* = 0.001; Emotional Regulation: b = −0.20, *p* < 0.001; Optimism: b = –0.38, *p* < 0.001; Self-efficacy: b = −0.21, *p* < 0.001; Social Support: b = −0.41, *p* < 0.001). Acute stress was a significant predictor of the resilience subscale self-efficacy (b = −0.16, *p* = 0.042). The number of major life events was a significant predictor of belongingness support (b = −0.14, *p* = 0.020) and the resilience subscale social support (b = 0.10, *p* = 0.031).

Neither family support nor belongingness support completely mediated any of the tested relationships. Family support partially mediated the relationship between chronic stress and the resilience subscale self-efficacy (Total effect = −0.25, Direct effect = −0.21, Indirect effect through family support = −0.03). Belongingness support partially mediated the relationships between chronic stress and the resilience subscale social support (Total effect = −0.45, Direct effect = −0.41, Indirect effect through belongingness support = −0.03). Similarly, belongingness support partially mediated the relationship between the number of major life events experienced in the past 12 months and the resilience subscale social support (Total effect = 0.06, Direct effect = 0.10, Indirect effect through belongingness support = −0.03). The remaining relationships were not mediated.

## 4. Discussion

The current study examined the relationships among stress, social support, and resilience in rural women living in the southeastern United States. The findings indicated that the participants had above-average levels of family support and slightly lower average levels of belongingness support. They also had above-average levels of resilience on all five subscales, including adaptability, emotional regulation, optimism, self-efficacy, and social support. A study objective was to examine whether stress was a predictor of resilience in younger, rural women. The results showed that their levels of acute and chronic stress were moderate. However, chronic stress predicted family and belongingness support and all the resilience subscales, while acute stress predicted the self-efficacy resilience subscale. Chronic stress was related to moderate stress levels regarding the physical environment and perceptions of safety in living spaces. The participants also reported having less stress related to the social environment and perceptions of unfair treatment by others.

The second study objective was to explore whether social support from family and friends, family support and belongingness support, mediated the relationship between acute stress and resilience and between chronic stress and resilience. Family support served as a partial mediator between chronic stress and self-efficacy as a resilience subscale. An explanation could be that having support from family can potentially increase confidence in circumventing aversity from stressful life events. Similarly, belongingness support served as a partial mediator between chronic stress and the social support resilience subscale. Perceptions of belongingness with friends and being in a group may enhance resilience and build social capital resources in limited resource areas. A related study outcome was that the women had above-average levels of stress regarding financial vulnerability. In fact, the majority (72.3%) of participants had difficulty paying for necessities such as food and shelter and required financial assistance in addition to their earned income. This finding raises questions about social policies that may perpetuate institutionalized economic exclusion of women living in rural areas.

The unexpected, yet interesting, outcomes were that the number of major life events predicted belongingness support and the social support resilience subscale, and in turn, belongingness support served as a partial mediator between the number of major life events and the social support resilience subscale. A possible explanation is that people reach out to friends and others in the rural community for support more frequently when enduring stress associated with traumatic major life events. It is also worth noting that the average number (approximately two) of major life events that occurred within the last 12 months is alarming given the seriousness of the events measured: serious illness or injury, victim of a serious attack, assault, or robbery, lost a loved one due to violence, loss of employment, serious illness, injury, death of a loved one, and divorce or separation from a spouse or partner. These results further highlight the urgent need to promote stress management strategies and social support resources among women living in rural areas. More research is needed among women, especially those having lower socioeconomic status, to explore whether social support as a downstream factor can simultaneously enhance resilience while concurrently reducing stress and impacting other modifiable cardiovascular disease risk factors [13,34].

Comparing the results of this study with other published research findings was challenging because the literature is scant. One study conducted among women living in rural areas of the southeastern United States indicated they had low chronic stress levels [51]. However, the findings of the current study done in approximately the same geographic location showed that rural women had higher to moderate levels of chronic stress. Another study conducted in this region showed that rural citizens had high levels of social support [52]. A few studies explored certain facets of stress, resilience, and social support among women in other regions. For example, a qualitative study collected the oral histories of women living in a rural area of New York and concluded that resilience was demonstrated by reliance on social support in response to stressors such as economic hardships and family life disruptions [53]. Another study among older adults in Singapore showed that social support was a mediator between resilience and caregiver burden [54]. The conclusions of a study among patients living with breast cancer in China were that greater levels of social support were associated with higher resilience levels and, interestingly, social support was a partial mediator between resilience and quality of life [55]. In comparison, social support did not predict resilience among elderly people in urban South America [56].

With the global aging of the general population, the implications for women’s health and nursing are to address the health care needs and preventive service challenges for the increasing numbers of older women [23]. There is promising evidence in empirical literature that intervention strategies can improve resilience [31,57,58,59]. Interventions designed to improve resilience may facilitate reductions in perceived stress [60]. Since stress is a risk factor for many chronic diseases, including cardiovascular disease, metabolic syndrome, and cancer, effective intervention strategies that help reduce stress among women and reduce chronic disease risk should be developed and implemented at the individual level through targeted chronic disease risk reduction programs. Primary prevention strategies can facilitate increasing the awareness of gender differences in emotional functioning regarding the physiological responses to stress by targeting sources of stress throughout the life trajectory [25]. Evidence-based stress management and reduction techniques can be added to augment the curriculum of such health promotion programs. For example, transcendental meditation is a strategy known to induce positive health effects, such as enhanced mood, reduced stress levels, and increased stress recovery [61].

At the societal level, the promotion of public health education strategies can increase awareness of the improved health effects experienced with adopting stress and stress-reduction techniques. Participatory research through community engagement can potentially affect structural changes that reduce stress risks and promote healthy lifestyles [62]. Community-based efforts that address environmental stressors could be implemented to circumvent some of the challenges associated with limited resources and opportunities found in rural areas, especially among low-income rural women [63]. Interventions that promote healthy lifestyles have a positive influence on physical health, promote factors associated with cardiovascular health among rural southeastern dwellers [64,65,66,67], and can mediate the relationship between socio-economic status and health [68,69]. However, the addition of strategies that foster individual, social, and environmental resources can facilitate resilience at the population level and buffer people from the negative health effects of stress [30]. Furthermore, public health policies should be enacted that address rural community needs, create assurance of services, and influence the allocation of governmental funding resources, such as federal block grant monies. Strategically transforming negative environmental factors in communities through focused interventions has the potential to promote well-being and reduce the visceral health effects of induced stress [62], which have been associated with cardiovascular disease. Social policies and evaluation of the policy impact on interventions designed to improve rural health outcomes are imperative to advance equity in the rural southeastern states.

The current study had some limitations. First, the cross-sectional design and data analysis methods used in the study does not allow for determining causal relationships or comparing outcomes among different groups. A longitudinal study would be needed to verify the mediating effect of social support on the association between stress and resilience. Second, the data collected on all measures were self-reported by participants and could suggest the potential for recall biases of some survey items and response bias to sensitive questions. Third, the study did not differentiate based on sociodemographic characteristics such as race, educational level, or marital status. Future research with larger sample sizes can facilitate testing the relationships among different groups and can detect disparities regarding health insurance, co-morbid medical conditions, and access to care. Fourth, the data were collected in the months immediately prior to the COVID-19 outbreak in the United States. Future studies could explore whether the pandemic induced a significant increase in stress, social support, and resilience factors in this population. Finally, the results of the current study may have limited generalizability to other regions. However, because stress, resilience, and social support are aspects of the human experience regardless of geographic location, the findings of this study can be compared with the results of similar rural studies throughout the world.

## 5. Conclusions

To our knowledge, this is the first study to provide insights into the relationships among stress, social support, and resilience in younger rural women living in the southeastern United States. Chronic stress is a threat to health and well-being, and research is needed to further explore this disease risk factor among rural populations. However, the biological mechanisms of chronic stress and linkages with female pathophysiology require innovative, gender-specific therapeutic treatments and stress management approaches. Interventions that foster existing and build new social support resources among rural women may facilitate the development of resilience that aids in counteracting stress. Social support from family and friends and belonging in social groups can help rural women buffer the stressful, and often unique, experiences encountered with daily living in remote areas. Such strategies may be crucial for reducing stress-related disease risk, promoting health, and advancing health equity in rural and remote populations.

## Figures and Tables

**Table 1:** Participant demographics.

Variable	Level	*n*	%
Marital Status	Single	239	67.5
	Married	81	22.9
	Other	34	9.6
Race	Black	289	81.6
	White	55	15.5
	Other	10	2.8
Ethnicity	Hispanic	11	3.1
	Not Hispanic	343	96.9
Highest Education	Some HS	64	18.1
	HS/GED	169	47.7
	Some College	102	28.8
	BS/BA or higher	16	4.5
Employment	Full-time	127	35.9
	Part-time	77	21.8
	Not employed	149	42.1
Children Under 18	Yes	244	68.9
	No	110	31.1
Financial Insecurity	Yes	256	72.3
	No	98	27.7
	Min, Max	M	SD
Age	18, 44	32.68	7.84
Number of Children	0, 8	1.52	1.52

**Table 2:** Standardized path coefficients, standard errors, and *p*-values for the fitted mediation model.

Dependent Variable	Independent Variable	Coefficient	Standard Error	*p*
Family Support	Chronic Stress	−0.19	0.06	0.001
Family Support	Acute Stress	0.01	0.02	0.455
Family Support	Major Life Events	−0.12	0.06	0.063
Belongingness Support	Chronic Stress	−0.12	0.06	0.035
Belongingness Support	Acute Stress	0.06	0.04	0.091
Belongingness Support	Major Life Events	−0.14	0.06	0.020
Resilience—Adaptability	Family Support	0.07	0.05	0.208
Resilience—Adaptability	Belongingness Support	0.09	0.06	0.108
Resilience—Adaptability	Chronic Stress	−0.18	0.05	0.001
Resilience—Adaptability	Acute Stress	−0.63	0.04	0.080
Resilience—Adaptability	Major Life Events	−0.03	0.05	0.628
Resilience—Emotional Regulation	Family Support	−0.02	0.07	0.789
Resilience—Emotional Regulation	Belongingness Support	0.01	0.06	0.897
Resilience—Emotional Regulation	Chronic Stress	−0.20	0.05	<0.001
Resilience—Emotional Regulation	Acute Stress	−0.12	0.07	0.111
Resilience—Emotional Regulation	Major Life Events	−0.01	0.05	0.898
Resilience—Optimism	Family Support	0.023	0.062	0.709
Resilience—Optimism	Belongingness Support	0.02	0.05	0.725
Resilience—Optimism	Chronic Stress	−0.38	0.05	<0.001
Resilience—Optimism	Acute Stress	0.03	0.04	0.449
Resilience—Optimism	Major Life Events	0.03	0.05	0.586
Resilience—Self-Efficacy	Family Support	0.15	0.06	0.018
Resilience—Self-Efficacy	Belongingness Support	0.11	0.06	0.064
Resilience—Self-Efficacy	Chronic Stress	−0.21	0.05	<0.001
Resilience—Self-Efficacy	Acute Stress	−0.16	0.08	0.042
Resilience—Self-Efficacy	Major Life Events	−0.01	0.06	0.916
Resilience—Social Support	Family Support	0.09	0.05	0.100
Resilience—Social Support	Belongingness Support	0.22	0.05	<0.001
Resilience—Social Support	Chronic Stress	−0.41	0.04	<0.001
Resilience—Social Support	Acute Stress	−0.08	0.05	0.116
Resilience—Social Support	Major Life Events	0.10	0.05	0.031

**Table 3:** Standardized effects—total direct and indirect effects for significant mediation relationships.

Relationship	Effect Type	Coefficient	Standard Error	*p*
Chronic Stress to Resilience—Self-Efficacy	Total Effect	−0.252	0.053	<0.001
	Total Indirect	−0.040	0.016	0.014
	Indirect through Family Support	−0.028	0.014	0.055
	Indirect through Belongingness Support	−0.012	0.009	0.162
	Total Direct	−0.212	0.054	<0.001
Chronic Stress to Resilience—Social Support	Total Effect	−0.448	0.044	<0.001
	Total Indirect	−0.042	0.016	0.010
	Indirect through Family Support	−0.017	0.010	0.094
	Indirect through Belongingness Support	−0.026	0.014	0.065
	Total Direct	−0.405	0.044	<0.001
Number of Major Life Events to Resilience—Social Support	Total Effect	0.063	0.050	0.208
	Total Indirect	−0.040	0.017	0.021
	Indirect through Family Support	−0.010	0.009	0.243
	Indirect through Belongingness Support	−0.029	0.015	0.045
	Total Direct	0.103	0.048	0.031

## Data Availability

The data presented in this study are available on request from the corresponding author. The data are not publicly available due to privacy issues.
